# Incidence of Induced Abortion and Post-Abortion Care in Tanzania

**DOI:** 10.1371/journal.pone.0133933

**Published:** 2015-09-11

**Authors:** Sarah C. Keogh, Godfather Kimaro, Projestine Muganyizi, Jesse Philbin, Amos Kahwa, Esther Ngadaya, Akinrinola Bankole

**Affiliations:** 1 Guttmacher Institute, New York, United States of America; 2 National Institute for Medical Research (NIMR), Dar-es-Salaam, Tanzania; 3 Muhimbili University of Health and Allied Sciences (MUHAS), Dar-es-Salaam, Tanzania; NHS lothian and University of Edinburgh, UNITED KINGDOM

## Abstract

**Background:**

Tanzania has one of the highest maternal mortality ratios in the world, and unsafe abortion is one of its leading causes. Yet little is known about its incidence.

**Objectives:**

To provide the first ever estimates of the incidence of unsafe abortion in Tanzania, at the national level and for each of the 8 geopolitical zones (7 in Mainland plus Zanzibar).

**Methods:**

A nationally representative survey of health facilities was conducted to determine the number of induced abortion complications treated in facilities. A survey of experts on abortion was conducted to estimate the likelihood of women experiencing complications and obtaining treatment. These surveys were complemented with population and fertility data to obtain abortion numbers, rates and ratios, using the Abortion Incidence Complications Methodology.

**Results:**

In Tanzania, women obtained just over 405,000 induced abortions in 2013, for a national rate of 36 abortions per 1,000 women age 15–49 and a ratio of 21 abortions per 100 live births. For each woman treated in a facility for induced abortion complications, 6 times as many women had an abortion but did not receive care. Abortion rates vary widely by zone, from 10.7 in Zanzibar to 50.7 in the Lake zone.

**Conclusions:**

The abortion rate is similar to that of other countries in the region. Variations by zone are explained mainly by differences in fertility and contraceptive prevalence. Measures to reduce the incidence of unsafe abortion and associated maternal mortality include expanding access to post-abortion care and contraceptive services to prevent unintended pregnancies.

## Introduction

With the 2015 Millennium Development Goals (MDG) deadline upon us, countries are evaluating their progress in reaching their targets. Despite achieving a 55% reduction between 1990 and 2015, Tanzania is falling short of its MDG5 target of cutting maternal mortality by three quarters, and continues to have one of the highest maternal mortality ratios in the world, at 410 per 100,000 live births [1]. In national consultations on the post-2015 development agenda, reducing maternal mortality was upheld as one of the ten key goals. Yet this cannot be achieved without addressing one of its leading causes, unsafe abortion.

Globally, unsafe abortions account for between 8% and 18% of maternal deaths [2,3], and millions more women suffer nonfatal health consequences of unsafe abortion every year [4]. In East Africa alone, an estimated 613,000 women were hospitalized for complications from induced abortion in 2005, or 10 per 1,000 women of reproductive age. Many more women suffer complications but do not access care: worldwide, an estimated one third of the 8.5 million women with abortion complications are not treated in facilities [4]. Global and regional estimates of abortion incidence indicate that abortions are no less common, and much more likely to be unsafe, in settings with restrictive laws than in settings with liberal laws [5]. Moreover, in countries where abortion is illegal or highly restricted, statistics are often not available, making it difficult to ascertain the magnitude of unsafe abortion and its consequences. In Tanzania, no nationally representative studies have been conducted to determine the incidence of abortion. However, according to regional estimates, in 2008 there were 2.4 million unsafe abortions, or 36 per thousand women of reproductive age, in East Africa [6].

The little evidence available for Tanzania, mainly from small-scale hospital-based studies, points to induced abortion being widespread, largely unsafe, and associated with high morbidity and mortality. According to these studies, just over 60% of women admitted to hospital for a miscarriage had in fact had an induced abortion [7–9]. Unsafe abortions accounted for 38% of hospitalizations for obstetric complications in one study [10], and roughly a quarter of maternal deaths in two other hospital studies [11,12].

The legal status of abortion is ambiguous in Tanzania: the Penal Code is broadly understood to authorize abortion to save a woman’s life, but remains unclear on its legality to preserve the woman’s physical or mental health [13]. Although Tanzania ratified the 2007 African Charter’s Protocol on the Rights of Women in Africa allowing abortion in cases of rape, incest or if the pregnancy endangers the woman’s life, mental or physical health or the life of the fetus [14], the government has not incorporated these provisions into its national law. There is also ambiguity over whether authorization is needed from more than one provider before performing an abortion [13,15]. This lack of clarity creates confusion amongst healthcare providers and women alike, and fear of prosecution on both sides pushes women to seek clandestine abortions that are often unsafe. As few people are willing to talk about abortion, very little information is available on the scope of the problem. This perpetuates the invisibility of unsafe abortion, resulting in the government giving it little priority in policy decisions, service delivery or program implementation.

In recent years, the Tanzanian government has shown strong commitment to reducing maternal mortality and morbidity through initiatives such as the National Road Map Strategic Plan to Accelerate Reduction of Maternal, Newborn and Child Deaths [16], and the approval of Misoprostol first for postpartum hemorrhage in 2007 and then for treatment of incomplete abortion in 2011 [17]. Building on a comprehensive post-abortion care (PAC) training program launched in 2000, the Tanzanian Ministry of Health and Social Welfare has since 2007 been expanding PAC services to lower level facilities in an effort to increase their availability throughout the country. However, still more needs to be done to ensure universal access to PAC. Many lower and mid-level facilities still lack equipment such as manual vacuum aspiration kits [18,19], and many hospital deaths following an abortion could be avoided with adequate training and staffing [12]. Efforts to expand access to comprehensive PAC require more evidence on the scale of induced abortion in Tanzania, both at the national and sub-national level, to determine where investments are most needed and how to allocate resources.

Reducing unsafe abortion also implies tackling its root cause, unintended pregnancy. The wanted total fertility rate (TFR), at 4.7 children per woman, remains significantly lower than the actual TFR of 5.4, due in part to low contraceptive use (25% amongst married women) [20]. In 2010, 26% of births were unplanned, representing a slight increase since 2005. Estimating the magnitude of unintended pregnancy, crucial for informing reproductive health programs and contraceptive service delivery, requires data on abortion incidence.

This paper presents findings from the first-ever nationally representative study measuring the incidence of abortion in Tanzania. This study responds to the express needs of in-country and international advocates working to promote reproductive health in Tanzania, who have highlighted the dearth of evidence on abortion as a major barrier to informed debate on the issue. This paper provides national and zonal estimates of the incidence of induced abortion, abortion complications and unintended pregnancy, with a view to informing strategies to reduce the country’s high maternal mortality.

## Materials and Methods

In countries where abortion is legally restricted and stigmatized, the approach to estimating abortion incidence must often be indirect. To overcome limitations in the availability of official statistics, the Guttmacher Institute has developed the Abortion Incidence Complications Methodology (AICM) [21] to estimate abortion incidence in these settings. The AICM has been applied in about 25 countries worldwide, including Senegal [22], Ethiopia [23], Burkina Faso [24], Uganda [25], Rwanda [26], Kenya [27], and Malawi [28] in sub-Saharan Africa. The methodology involves estimating the number of induced abortion complications treated in facilities, and using expert opinions to estimate, for each complication that reaches a facility, how many induced abortions are occurring without complications or with untreated complications.

The primary data for this study come from two surveys conducted by the authors: a Health Facilities Survey (HFS) to measure the number of abortion complications treated in health facilities, and a Health Professionals Survey (HPS) to estimate the likelihood of women experiencing abortion complications and of obtaining treatment at a health facility. The study design and protocols are adapted from previous applications of the methodology, to be relevant to the Tanzanian context. Data collection was led by the Tanzanian National Institute for Medical Research (NIMR) in Dar-es-Salaam, with technical support from Muhimbili University of Health and Allied Sciences (MUHAS) and the Guttmacher Institute. The data from the two surveys were used together with estimates of births, unintended births, and women of reproductive age, compiled from the 2010 Tanzania Demographic and Health Survey [20] and the 2012 Tanzanian national census [29] and projected to 2013 using Tanzania's intercensal annual growth rate. Estimates were obtained at the national level, as well as for each of the 8 geopolitical zones of Tanzania (7 in mainland, plus the semi-autonomous Zanzibar archipelago).

Fieldwork was conducted from July to September 2013. Interviewers were recent medical graduates with previous experience administering surveys. For the HFS, a team of four interviewers was assigned to each zone, and members of the research team at NIMR acted as team leads. These team leads also conducted the HPS interviews, as the HPS questionnaire required more skill and experience, and many respondents were senior professionals. All interviewers and team leads underwent a week-long training in Dar-es-Salaam. Both surveys were piloted in 5 facilities which were not part of the final sample.

### Heath Facilities Survey

The sampling frame consisted of the Ministry of Health’s most recent list of all health facilities (public and private) considered likely to provide post-abortion care (PAC). A separate list of all public and private facilities was obtained from the Zanzibar Ministry of Health. Facilities that did not provide primary care, that were specialized in non-reproductive services, or that otherwise lacked the capacity to provide PAC (as for some dispensaries), were excluded from the sampling frame. To ensure that the list was up to date, all district medical officers in the country were contacted to confirm that all health facilities in their district were included in the list, and to determine which facilities were equipped to provide PAC.

The final sampling frame of facilities likely to provide PAC included 952 facilities, of which 5 (0.5%) were consultant hospitals, 20 (2.1%) regional hospitals, 224 (23.6%) sub-regional hospitals (district hospitals or other hospitals), 526 (55.2%) health centers and 177 (18.6%) dispensaries (Table 1). Although there were thousands of dispensaries in Tanzania, only 177 provided or were expected to provide PAC at the time we conducted the study. The nomenclature for facilities in Zanzibar is slightly different, with Primary Health Care Centers (PHCCs) being the equivalent of health centers, and Primary Health Care Units (PHCUs) being the equivalent of dispensaries. Within PHCUs, some provide only basic primary care services (PHCU), while others are equipped to provide a wider range of services including PAC (PHCU+). PHCCs and PHCU+ were combined with health centers and dispensaries respectively in the national level analyses.

**Table 1:** Characteristics of sample, by facility type, Health Facilities Survey, Tanzania 2013.

	Number of facilities likely providing PAC	Number selected (sampling proportion)		Number responded (response rate)		Number providing PAC by ownership			
						Public	Private	Faith-based organization	Total
**Facility level**									
Consultant hospital	5	5	(100%)	5	(100%)	3	0	2	**5**
Regional hospital	20	20	(100%)	20	(100%)	18	0	2	**20**
Non-regional hospital	224	149	(66%)	144	(97%)	60	23	59	**142**
Health center	526	235	(45%)	234	(100%)	181	9	18	**208**
Dispensary	177	78	(44%)	78	(100%)	68	2	3	**73**
**Total**	**952**	**487**	**(51%)**	**481**	**(99%)**	**330**	**34**	**84**	**448**

The sampling frame was stratified by facility level within each zone, to ensure that the estimates of abortion incidence were representative at both the national and zonal levels. Within each zone, we selected 100% of consultant and regional hospitals, 66% of non-regional hospitals, 45% of health centers, and 44% of dispensaries equipped to offer PAC, resulting in a total of 487 health facilities in the initial sample. Of these, 481 agreed to participate in the survey (Table 1). To minimize refusals, letters from the Ministry of Health introducing and authorizing the study were sent to all facilities in advance of the fieldwork, and facility in-charges were contacted to inform them about the upcoming survey. Of the facilities that responded, 448 (93%) actually provided PAC services. Most health facilities in Tanzania are government-owned, and this is reflected in the sample: only 26% of all facilities (mostly hospitals) were private or faith-based (Table 1).

A structured questionnaire was administered by an interviewer to the most qualified staff member or the person in charge of providing PAC in each facility, typically the chief of the Obstetrics and Gynecology department in larger facilities, and the facility head in health centrs or dispensaries. Respondents were asked whether their facilities provide treatment for abortion complications (from both spontaneous and induced abortion), and if so, to estimate the number of PAC patients treated as outpatients and inpatients, in an average month and the past month. Specifying two time frames increases the likelihood of capturing variation from month to month. These two numbers were subsequently averaged and multiplied by 12 to produce an estimate for the 2013 calendar year.

### Health Professionals Survey

The Health Professionals Survey interviewed a purposive sample of 202 experts knowledgeable about abortion provision in Tanzania. The sample was created through consultation with a broad network of colleagues engaged in research, policy, community and regional-level public health programs, which aimed to identify the individuals most knowledgeable about the provision of abortion at the national level, as well as in each zone. The experts came from a wide range of professions. Forty-six percent were health professionals, including obstetricians/gynecologists, midwives and nurses from the public and private sector, as well as a sizeable proportion of non-formally trained health workers such as community health workers, traditional birth attendants and traditional healers (15% of total sample). The remaining 54% of the sample was composed of researchers, reproductive health advocates, non-governmental organization (NGO) and women’s groups’ representatives, lawyers, journalists working on reproductive health issues, program managers and policy makers, community leaders, and youth leaders. Respondents were distributed equally between the 8 zones. Particular effort was made to ensure that there was sufficient representation of experts with knowledge of rural areas. About 36% of respondents had worked at least 6 months in rural areas during the last 5 years. All 202 prospective respondents agreed to participate. Questionnaires sought information on the proportion of women who experience a complication from an induced abortion, and the proportion likely to receive care in a facility should they experience a complication, separately for rural and urban, poor and non-poor women.

### Ethics statement

Ethical approval was obtained from Guttmacher’s Institutional Review Board, the Tanzanian Medical Research Coordinating Committee, and Zanzibar Medical Research Council. All respondents in both surveys gave written informed consent before being interviewed.

### Data analysis

From the Health Facilities Survey (HFS), we obtained estimates of the number of patients treated for abortion complications nationally and for each zone. To ensure estimates were representative at the national and zonal levels, weights were assigned to each facility by level and zone, based on their selection probability and on non-response rates. To avoid double-counting patients treated at one facility level then referred to another for additional treatment, we subtracted 75% of patients treated then referred at each facility level from the number treated at the next level, based on an informed estimate that roughly 75% of patients referred for obstetric complications follow up in Tanzania (urban and rural settings combined) [30]. Due to the similarity between complications from induced and spontaneous abortions, and the possibility of patients and/or providers misreporting induced abortions as spontaneous for fear of legal sanctions, it is difficult to obtain accurate estimates of complications solely from induced abortions at the facility level. Therefore the survey recorded the number of complications from all abortions (both induced and spontaneous), from which we then subtracted those due to spontaneous abortion, to obtain the number of induced abortion complications treated in health facilities, nationally and by zone [21]. The number of spontaneous abortion complications treated in facilities was calculated using indirect estimation techniques. Assuming that only second trimester spontaneous abortions require care, and that these equal 3.41% of live births based on clinical studies [31,32], we obtained the number of spontaneous abortions that would need treatment nationally and by zone. However, not all spontaneous abortions requiring care will actually be treated in facilities for a number of reasons, including lack of access or a preference to seek treatment from untrained providers. To estimate the proportion of spontaneous abortions needing care that were actually treated in health facilities in each zone, we assume that this proportion is similar to the proportion of recent births that were either delivered in health facilities or not delivered in health facilities because it was not customary or necessary. The 2010 DHS estimate was projected to 2013 based on percent change between the last two DHS rounds) and adjusted it to include women who would have delivered in health facilities had it been customary or necessary using DHS information on women’s reasons for not delivering at facilities.

But not all induced abortions will result in treated complications. Some will be done without complications, while others will end in complications that are not treated in health facilities for various reasons, including lack of access, fear of prosecution, preference to seek treatment from untrained providers or even death. The HPS provided estimates of the probability of experiencing induced abortion complications by type of abortion provider, and the probability of seeking care for complications, for four wealth-residence groups: urban poor, urban non-poor, rural poor and rural non-poor. Multiplying these two sets of probabilities, we obtained the proportion of all induced abortions that resulted in complications that were treated, for each of the four groups. These were then combined into a single weighted proportion based on the population distribution of the four groups (from the DHS), nationally and separately for each zone. The inverse of this proportion is the multiplier or inflation factor needed to account for induced abortions which were either without complications or with complications that were not treated in a facility. This multiplier presents the number of such abortions for every induced abortion complication treated in a facility. The higher the multiplier, the higher the proportion of abortions that is either uncomplicated or with untreated complications [21]. We multiplied this factor by the number of induced abortion complications treated in health facilities (from the HFS), to obtain the total number of induced abortions nationally and for each zone in 2013 [21]. The number of induced abortions is expressed per 1,000 women aged 15–49 (abortion rate) and per 100 live births (abortion ratio).

We also calculated the number and rates of total pregnancies and unintended pregnancies nationally and for each zone. The total number of pregnancies is calculated as the sum of the annual numbers of induced abortions, births and miscarriages (estimated as 20% of live births plus 10% of induced abortions [31,33]). Expressing this number per 1,000 women of reproductive age gives the pregnancy rate for each zone and nationally. The total number of unintended pregnancies is the sum of induced abortions, unplanned births (obtained by multiplying the proportion of recent births that were unintended in the DHS by the total number of births in 2013), and unplanned pregnancies resulting in miscarriage (equal to 20% of unplanned births plus 10% of induced abortions, assuming that all induced abortions result from unintended pregnancies [33]). This estimate can be expressed per 1,000 women of reproductive age to obtain the unintended pregnancy rate.

## Results

### Abortion treatment rates, incidence rates and ratios

There are just under 8 facilities providing PAC per 100,000 women in Tanzania (Table 2). Accessibility of PAC varies across the country: Zanzibar is the best served with over 10 facilities per 100,000 women, while the Eastern zone had the lowest density, with under 6 facilities per 100,000 women. Consultant and regional hospitals treat the majority of Tanzania’s post-abortion care cases. In 2013, on average, each consultant and regional hospital treated about 1,140 and 710 PAC cases, respectively, compared to average annual caseloads of about 250 at each sub-regional hospital, 70 at each health center, and less than 30 at each dispensary. Overall, about 66,640 women were treated in facilities for complications from induced abortions, at a rate of 5.9 per 1,000 women age 15–49 (Table 3). This rate varied considerably by zone, from a low of 2.9 induced abortion cases treated per 1,000 women in the Eastern zone to a high of 7.9 per 1,000 in the Southern Highlands.

**Table 2:** Number of facilities providing PAC, and average caseload, by zone and nationally, Tanzania 2013.

		Central	Eastern	Lake	Northern	Southern	Southern Highlands	Western	Zanzibar	Total (national)
**Number of facilities providing PAC** ^**a**^										
Total		65	134	199	155	64	104	121	37	877
Per 100,000 women		8.2	5.8	8.4	9.2	7.2	7.1	8.8	10.5	7.8
**Annual average PAC caseload by facility type**										
*Level*										
Consultant hospital		N/A	174	1,620	330	N/A	2,640	N/A	930	1,139
Regional hospital		912	531	1,144	580	442	670	720	N/A	707
Sub-regional hospital		216	216	410	216	166	173	355	170	247
Health center		42	101	91	60	48	93	68	19	72
Dispensary		N/A	38	18	20	N/A	N/A	35	20	26
*Ownership*										
Public		174	137	130	126	132	236	99	107	140
Faith-based organization		103	230	485	210	120	131	436	12	254
Private		N/A	156	84	99	N/A	89	118	N/A	128

^a^ Adjusted to discount facilities "likely providing PAC" that do not in fact provide PAC. Facilities in the initial sample that were found upon surveying to not provide PAC were proportionally subtracted from the universe of facilities likely providing PAC, separately for each zone.

**Table 3:** Number of women treated for abortion complications, by zone and nationally, Tanzania, 2013.

	Women aged 15–49	Women treated for abortion complications	Women treated for miscarriages in facilities	Women treated for induced abortions in facilities	Rate of treatment for induced abortion complications
**Zone**					
Central	789,106	6,772	3,024	3,748	4.7
Eastern	2,289,015	16,106	9,398	6,708	2.9
Lake	2,382,254	28,408	10,354	18,054	7.6
Northern	1,683,655	18,009	5,106	12,903	7.7
Southern	876,956	6,343	2,661	3,682	4.2
Southern Highlands	1,470,700	16,079	4,444	11,635	7.9
Western	1,370,458	13,986	5,116	8,870	6.5
Zanzibar	348,499	2,305	1,264	1,041	3.0
**Total (national)**	11,210,642	108,008	41,367	66,641	5.9

Women obtained approximately 405,000 induced abortions in Tanzania in 2013, for a national rate of 36 abortions per 1,000 women age 15–49 and a ratio of 21 abortions per 100 live births (Table 4). The national multiplier is 6.08, meaning that for each woman treated in a facility for induced abortion complications, 6 times as many women had an abortion but did not receive PAC–either because they did not experience complications, or because their complications went untreated. Both abortion incidence rates and multipliers vary widely by zone. Zanzibar has the lowest multiplier, 3.6, and the lowest abortion rate, 10.7 (Table 4). The Lake zone has by far the highest abortion rate, 51, while the Eastern zone has the highest multiplier, 8.

**Table 4:** Estimates of induced abortion, by zone and nationally, Tanzania, 2013.

	Estimated total number of induced abortions	Abortion rate (per 1,000 women 15–49)	Abortion ratio (per 100 live births)	Multiplier
**Zone**				
Central	21,923	27.8	13.6	5.85
Eastern	54,655	23.9	18.2	8.15
Lake	120,857	50.7	25.0	6.69
Northern	51,965	30.9	22.2	4.03
Southern	23,465	26.8	19.2	6.37
Southern Highlands	68,910	46.9	27.0	5.92
Western	59,592	43.5	19.3	6.72
Zanzibar	3,714	10.7	7.1	3.57
**Total (national)**	**405,081**	**36.1**	**21.1**	**6.08**
*Lower bound*	*282*,*588*	*25*.*2*	*14*.*7*	
*Upper bound*	*527*,*573*	*47*.*1*	*27*.*5*	

The 95% confidence interval for the national number of induced abortion complications treated in facilities was multiplied by the national multiplier to obtain lower and upper bounds for the number of abortions and national abortion rate. The lower and upper bounds for the national abortion rate were 25 and 47 (Table 4). Because the number of facilities in each zone was too low to obtain robust confidence intervals of PAC cases, we only present this information at the national level.

### Unintended pregnancy rate

The number of induced abortions was used to calculate pregnancy rates for each zone and nationally (Table 5). The pregnancy rate for Tanzania is 245 per 1,000 women of reproductive age, with wide variations by zone from 184 in the Eastern zone to 318 in the Western zone. The unintended pregnancy rate is somewhat lower at 92.7 per 1,000 women of reproductive age, ranging from 61.5 in Zanzibar to 123.8 in the Lake zone.

**Table 5:** Unintended births, pregnancies, and pregnancy rate, by zone and nationally, Tanzania 2013.

	Total Fertility Rate ^a^	Wanted Total Fertility Rate ^a^	% women 15–49 sexually active in last 4 weeks ^a^	% women 15–49 who have never had sexual intercourse^a^	% sexually active women using any contraceptive method ^a^	% sexually active women using modern methods ^a^	% women with unmet need for contra-ception ^a^	Total preg-nancies ^b^	Total unintended preg-nancies ^c^	Pregnancy rate (per 1,000 women)	Unintended pregnancy rate (per 1,000 women)

**Zone**											
Central	6.5	5.7	58.2	13.0	35.4	33.6	26.7	217,645	81,532	275.8	103.3
Eastern	3.9	3.6	55.3	11.3	51.4	41.6	13.0	421,092	160,257	184.0	70.0
Lake	6.3	5.1	62.9	12.3	18.5	16.8	25.1	712,069	295,033	298.9	123.8
Northern	4.6	3.8	54.7	18.0	54.0	44.4	16.1	338,511	116,342	201.1	69.1
Southern	4.4	4.0	61.1	7.7	51.8	39.5	17.1	172,384	62,400	196.6	71.2
Southern Highlands	5.4	4.7	56.3	14.7	48.8	36.5	15.1	381,564	116,303	259.4	79.1
Western	7.1	6.3	57.5	13.5	24.4	19.3	18.9	435,603	133,992	317.9	97.8
Zanzibar	5.1	4.8	48.5	33.8	19.7	14.5	20.7	66,771	21,408	191.6	61.4
**Total (national)**	**5.4**	**4.7**	**55.3**	**13.8**	**35.6**	**29.7**	**19.1**	**2,745,637**	**1,039,001**	**244.9**	**92.7**

^a^ Obtained from the 2010 Tanzanian DHS

^b^ Sum of estimated number of abortions (from this study) and estimated number of births and miscarriages (from the DHS).

^c^ Sum of estimated number of abortions (from this study) and estimated number of unintended births and miscarriages (from the DHS).

**Fig 1:**
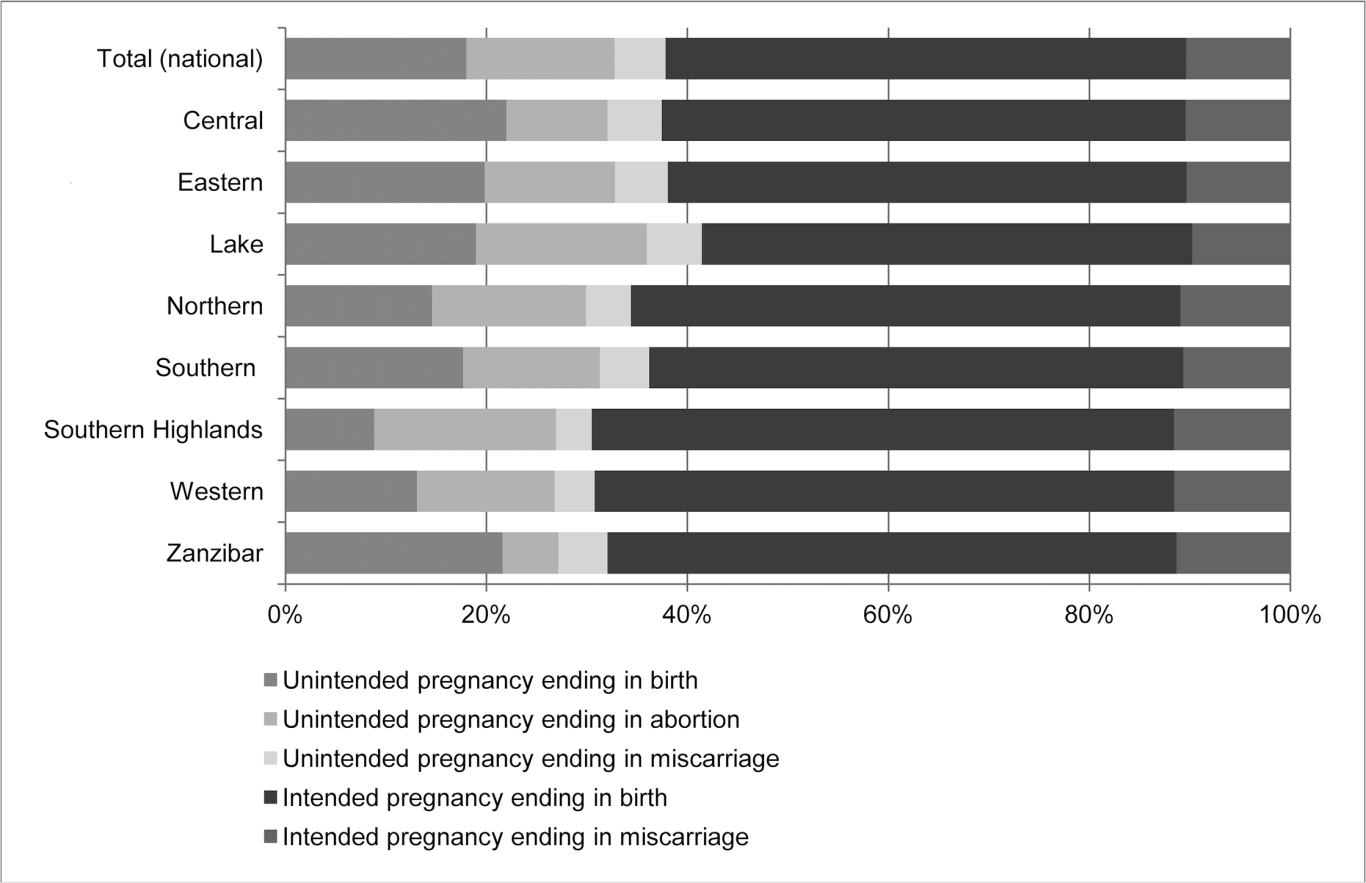
Distribution of pregnancy outcomes, nationally and by zone, Tanzania 2013. In Tanzania in 2013, 15% of all pregnancies ended in abortions, 52% in intended births, 18% in unintended births, and 15% in miscarriages. The distribution of pregnancy outcomes varied across zones: in the Lake zone, 41% of pregnancies were unintended, and 17% ended in abortion. In contrast, in Zanzibar only 32% of pregnancies were unintended, and 6% ended in abortion.

The data on unintended pregnancies, births, abortions, and miscarriages were used to estimate the distribution of pregnancies by outcome and intention status. In the country as a whole, 15% of pregnancies ended in abortions, 52% in intended births, 18% in unintended births, and 15% in miscarriages (Fig 1). These distributions vary across zones; for example, the percentage of total pregnancies ending in abortion ranges from 6% in Zanzibar to 18% in Southern Highlands.

## Discussion

The 2013 abortion rate for Tanzania (36 abortions per 1,000 women of reproductive age) is the same as the 2008 rate estimated by WHO for East Africa as a whole [6], suggesting Tanzania is representative of the region. The latest national-level estimates for neighboring countries using the AICM indicate Tanzania’s rate is lower than Kenya’s (48 per 1,000), similar to Uganda’s (37), and higher than the rates for Ethiopia (23), Rwanda (25) and Malawi (24) [25–28]. Although estimates for Ethiopia, Rwanda, Malawi and Uganda are for women aged 15–44 rather than 15–49, the rankings remain unchanged if estimates use the same denominator.

The wide variations by zone in the estimates of abortion incidence, complications and treatment reflect differences in access to health services, as well as demographic and cultural differences. The highest abortion rates and ratios are found in the Lake Zone and Southern Highlands, where rates of treatment for induced abortion complications are also highest. In the Lake zone, the high abortion rate is most likely a consequence of low contraceptive use (lowest in the country) and high unmet need, which are also responsible for the highest unintended pregnancy rate in the country. In contrast, in the Southern Highlands where contraceptive use is high and unmet need relatively low, the high abortion rate is explained by the fact that women are more likely to resort to abortion to end an unintended pregnancy than in other zones: 59% of unintended pregnancies ended in abortion in Southern Highlands, compared to less than 45% in other zones. Consequently, the Southern Highlands have the lowest proportion of unintended pregnancies ending in birth, and the lowest rate of unplanned births.

Zanzibar’s low abortion incidence rate, at 11 per thousand, is mainly due to it having the lowest unintended pregnancy rate in the country (61 per thousand). However, this has not been achieved through high contraceptive use, since Zanzibar has the lowest modern-method contraceptive prevalence (14.5%) and second lowest all-method prevalence in Tanzania. Instead, unintended pregnancy rates may be lower due to reduced sexual activity: Zanzibar has the lowest proportion of women age 15–49 who were sexually active in the last 4 weeks (49% vs. 58% in Mainland overall) and the highest proportion of women who have never had sexual intercourse (34% vs. 13%). Women in Zanzibar also tend to initiate sexual activity later (at a median age of 19.2 years) than their Mainland counterparts (median age of 17.4) [20]. Zanzibar being 98% Muslim, extramarital fertility and sex outside of marriage are taboo [34], which may contribute to explaining these patterns. Higher proportions of women in polygynous relationships in Zanzibar (29%) compared to the Mainland (21%) may also contribute to lower sexual activity levels, although evidence to support this hypothesis is lacking.

The Western zone, which has the highest pregnancy rate, also displays the highest wanted TFR in the country (6.3), and has relatively low contraceptive use. The Western zone's unintended pregnancy rate, on the other hand, is relatively low due to high desired fertility. The lowest pregnancy rate in the Eastern Zone is consistent with the observed lowest desired and actual fertility and highest contraceptive use in the country.

The regional differences in treatment rates for complications, while partly reflecting differences in abortion incidence rates, also highlight the unequal distribution of PAC providers throughout the country, with some regions much better served than others. This underscores the importance of investing in PAC at all levels of the health system. While Zanzibar has successfully expanded its PAC program to all Primary Health Care Units (PHCU+), initiatives to decentralize PAC provision to lower level facilities in Mainland have not yet been rolled out in all regions. These efforts should be strengthened: the moderate uptake of PAC at the dispensary level (averaging 26 cases per facility in 2013), well below the average uptake in health centers (72 cases), suggests more needs to be done to ensure dispensaries are fully equipped to provide basic PAC, including training midlevel providers and ensuring facilities are adequately stocked with necessary drugs, supplies and equipment, at all levels. Manual vacuum aspiration (MVA) kits are currently missing from many facilities [18], and despite Misoprostol’s high acceptability for women in Tanzania [35] and recent approval for treatment of incomplete abortion [17], very few facilities currently stock it or have providers trained to use it [18]. Even if lower-level facilities are fully equipped, women may be unaware of the services offered or unsure of their quality. In parallel to expanding PAC services, increased efforts should be made to reach women through multiple avenues with information about types of PAC services available at each facility level.

Ensuring universal access to PAC will also help meet contraceptive demand, since post-abortion patients are very receptive to contraceptive counseling [19,36,37], and have high method acceptance rates (around 90%) and continuation rates (86% when interviewed 1–6 months post-abortion, around 80% at 12 months) [38,39]. Men accompanying their partners for PAC were also found to be receptive to contraceptive information at that time, suggesting PAC services may provide a valuable opportunity to reach men with contraceptive information [40]. Increased contraceptive use will in turn decrease the need for unsafe abortion, since unintended pregnancy remains a major cause of abortion and most unintended pregnancies are due to non use of contraception [41]. According to a 2012 study, meeting contraceptive demands in sub-Saharan Africa would reduce unintended pregnancies by 78% [42]. Integrating family planning services into pre and post-abortion care and ensuring that they offer a wide range of methods along with contraceptive counseling, has the added benefit of reaching women at increased risk of unintended pregnancy and unsafe abortion with the information and services they need to prevent unintended pregnancies. These steps can further increase the potential impact of investing in integrated PAC and contraceptive services.

## Conclusion

This is the first study to provide nationally representative estimates of the incidence of induced abortion in Tanzania. In a country where unsafe abortion is a major driver of high levels of maternal mortality, this study provides pertinent, timely and reliable evidence to inform policy debates at a time when public engagement and action around abortion and post-abortion care is gathering pace. Tanzania has shown commitment to improving maternal health in recent years. The post-2015 development agenda provides an opportunity to come up with cost-effective and targeted action plans to fulfill these commitments. Improving access to contraception to prevent unintended pregnancies, many of which end in abortions, and investing in universal access to PAC, including post-abortion contraceptive services, while prioritizing areas with the highest abortion rates such as the Lake zone and Southern Highlands, constitute a cost-effective and multi-faceted approach to reducing maternal mortality and morbidity, unintended pregnancy and unsafe abortion, with far-reaching impacts for the well-being of women and their families in Tanzania.
